# Using Support Vector Machines to Classify Road Surface Conditions to Promote Safe Driving

**DOI:** 10.3390/s24134307

**Published:** 2024-07-02

**Authors:** Jaepil Moon, Wonil Park

**Affiliations:** 1 Department of Highway & Transportation Research, Korea Institute of Civil Engineering and Building Technology, Goyang 10223, Republic of Korea; 2 Department of Civil & Environmental Engineering, Seoul National University, Seoul 08826, Republic of Korea

**Keywords:** road surface condition, traffic safety, adverse winter weather, data-driven learning model, SVM, linear classifier, nonlinear classifier, posterior probability

## Abstract

Accurate detection of road surface conditions in adverse winter weather is essential for traffic safety. To promote safe driving and efficient road management, this study presents an accurate and generalizable data-driven learning model for the estimation of road surface conditions. The machine model was a support vector machine (SVM), which has been successfully applied in diverse fields, and kernel functions (linear, Gaussian, second-order polynomial) with a soft margin classification technique were also adopted. Two learner designs (one-vs-one, one-vs-all) extended their application to multi-class classification. In addition to this non-probabilistic classifier, this study calculated the posterior probability of belonging to each group by applying the sigmoid function to the classification scores obtained by the trained SVM. The results indicate that the classification errors of all the classifiers, excluding the one-vs-all linear learners, were below 3%, thereby accurately classifying road surface conditions, and that the generalization performance of all the one-vs-one learners was within an error rate of 4%. The results also showed that the posterior probabilities can analyze certain atmospheric and road surface conditions that correspond to a high probability of hazardous road surface conditions. Therefore, this study demonstrates the potential of data-driven learning models in classifying road surface conditions accurately.

## 1. Introduction

As slippery road surfaces owing to snow or ice constitute a major cause of fatal car accidents, accurate detection of road surface conditions in adverse winter weather is essential for traffic safety. According to a press release from the Samsung Traffic Safety Research Institute (2020) [1], the average annual numbers of traffic accidents and fatalities due to icy roads during the five years from January 2014 to December 2018 in South Korea were 1310 and 40, respectively, with the fatality rate being 1.6 times higher than the average for all traffic accidents per 100 incidents. Furthermore, reports show that there is a strong correlation with ice-related accidents when the minimum temperature is below 0 °C and the daily temperature ranges exceed 9 °C.

To prevent traffic accidents caused by icy roads and to enhance winter road services, road management agencies install and operate safety equipment and signs, such as road heating, automatic saltwater spraying devices, and safety markings, in areas prone to icing. In addition to the installation of these safety facilities and signs, efforts are being made to monitor and detect icy roads much earlier to provide drivers with relevant information.

Research has been conducted to predict road surface conditions using various sensors installed on vehicles, at the roadside, or through the roadway weather information system (RWIS). A common method to determine road surface conditions is to monitor images through CCTV or to patrol the roads within the area daily, measuring road surface temperatures and using visual inspection. However, the manpower and equipment used in these road monitoring and patrol methods are inefficient. Moreover, as advanced detectors installed on vehicles or at the roadside for accurate road surface condition assessments are costly, it is impossible to install such detectors on national roads. In addition, each of the detectors predicts the road surface condition by means of a classification model based on its own specific data, and then the accuracy of the detectors may be affected by external environments.

Therefore, from the perspective of occupant safety and cost, as well as winter road maintenance, there is a need to balance safety with limited resources while presenting the budget for the annual winter challenge. Given the limited road maintenance personnel and budget constraints, an efficient data-driven approach to road management is proposed in this study to predict icy conditions. This approach involves a model to classify road surface conditions using low-cost detectors and open-source data, such as road surface temperature and weather information from the Korean Meteorological Administration. Using a data-driven road surface condition classifier to assess the likelihood of surface ice formation for safe driving, road patrols, and road maintenance tasks, like snow removal agent applications, can improve effectiveness and responsiveness. In addition, the information would effectively and prominently improve the management of road safety in conjunction with index modulation multiple access (IMMA) [2] as a promising sixth-generation (6G) technique, or spatial modulation (SM) [3] as a new transmission technology for maximizing the throughput and minimizing the cost.

This paper is structured as follows. First, the existing literature is reviewed to examine ways to enhance the accuracy and reliability of road surface condition predictions and analysis methods. We then report on the analysis methodology that was established and applied to train and test the machine learning model using preprocessed data from onsite observations. Finally, the established learning model is evaluated in terms of the generality and reliability of road surface condition estimation.

## 2. Literature Review

Abdic et al. [4] developed a model that detects wet and dry road surface conditions by training a recurrent neural network using tire noise data from vehicles according to pavement conditions; this model included variables such as vehicular speed, ambient environmental noise, road pavement type, and the friction coefficient. This model achieved approximately 93% accuracy in predicting road surface conditions based on vehicular speeds, noises from the environment, road surface types, and pavement conditions.

Nolte et al. [5] applied two deep convolutional networks to predict road surface conditions and evaluated the friction coefficients (which are directly correlated with road surface conditions) based on the image data of a road surface. The accuracy of their test data was 92%. However, the road surface conditions were limited to wet and dry, and they focused on asphalt, cement, and blacktop road pavement types.

Pan et al. [6] used a pretrained deep convolutional network model to develop a road surface condition evaluation model and applied it to road surface image data collected via smartphones. They enhanced the pretrained model by adding two fully connected layers of neurons to extract features from the image data and achieved a classification performance comparable to that of conventional machine learning models.

Carrillo et al. [7] developed a road surface condition prediction model by applying seven pretrained deep learning models (DenseNet, NASNet, etc.) for efficient winter road management to determine the snow removal priorities based on road surface conditions. The proposed model can determine road surface conditions in situations with poor visibility using road surface images collected from roadside cameras and weather information (ambient temperature, wind speed, pressure, humidity, and dew point) acquired simultaneously from RWIS. However, as this study aimed to determine snow removal priorities, the road surface conditions considered for machine learning were limited to the level of snow coverage.

Seo et al. [8] proposed a spatiotemporal structure that uses a deep neural network to classify road pavement conditions (dry, wet, snow) and types (asphalt, cement, gravel, blacktop) using light detection and ranging (LiDAR). The explanatory variables considered for machine learning included average reflectivity and the number of point clouds from the LiDAR sensor, as well as vehicular speed, which affects LiDAR data. The accuracy of their model was approximately 98%.

Most of the previous studies have focused on the classification of road surface conditions and pavement types based on image data or specific detectors. Furthermore, in the case of classification of road surface conditions associated with specific detectors, data related to vehicle behaviors (e.g., speed (Seo et al. [8])) may also be required. Therefore, this study differs from existing studies in that it builds a road surface condition classification model based on road surface and atmospheric weather information collected from low-cost roadside detectors or open sources, rather than applying a road surface condition classification model based on data collected from specific detectors. In addition, it is less affected by the road environment compared to models based on image data or data collected from expensive equipment.

## 3. Study Methodology

This study presents a data-driven learning model with high accuracy and generalizability that considers readily accessible meteorological data (for example, ambient or road surface temperature and humidity) from an open-source RWIS using low-cost equipment. We selected the support vector machine (SVM), which has been successfully applied to solve classification problems in diverse fields. Although other machine learning models are available for classification, the purpose of this study was not to develop a model with superior predictive performance, but rather to demonstrate the feasibility of accurate road surface condition prediction using data-driven learning models.

The toll expressway periodically patrols the road patrol car for each route for road safety management during the snow removal period set by the Korea Expressway Corporation. If the road surface is likely to be slippery or icy due to the weather forecast, a specific detector is attached to check the road surface condition during patrol on the planned patrol route map. Besides the confirmation of the road surface state, atmosphere- and road surface-related weather information are collected together at regular intervals during patrolling between the set origin and terminal of each patrol route. This study was carried out with the data collected during the winter patrol.

**Figure 1:**
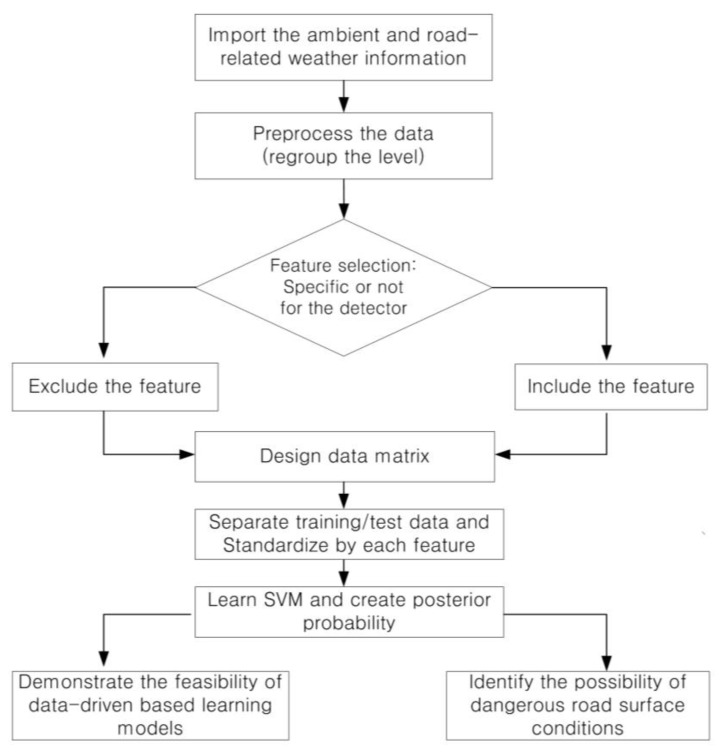
Study performance flowchart.

As shown in Figure 1, first of all, we select items that can be collected or produced from low-cost detectors or open sources among the data provided by the expressway institute. The selected items are applied as the features of a data-driven learning model, with the corresponding road surface state as its target label. The preprocessed data are randomly allocated in some ratio to training data and test data groups. Before feeding into a learning model, data standardization is processed to convert different scales into a uniform scale across features, ensuring that some features do not dominate others due to their magnitude. The standardization is re-scaled by the Z-Score method. As mentioned above, the SVM is applied not to develop an accurate model, but to evaluate the predictability of data-driven learning models for classifying road surface conditions. The SVM learns via the training data, and both the linear and nonlinear methods are applied, with soft margin classification and kernel functions. Finally, a confusion matrix is applied for a generalization performance using the test data, and it determines the feasibility of the accurate road surface condition predictions of data-driven learning models based on the results of this study. In addition to evaluating the feasibility of data-driven learning models, the posterior probability belonging to each road surface condition is separately calculated to identify the possibility of dangerous road surface conditions in certain environments. The classification scores obtained from the trained SVM are applied to the sigmoid function, and the posterior probability of each road surface condition is produced.

## 4. Learning Data

### 4.1. Data Collection

Data related to weather and road surface temperature for training and testing the SVM were collected from 26 expressways nationwide between December 2022 and March 2013. These data were collected at intervals of 0.2 s using high-cost mobile advanced road weather information sensor (MARWIS) equipment mounted on a vehicle while driving through each section. MARWIS, which uses optical light-emitting diode transmitters, photo-receivers, pyrometers, and infra-red technology, collected data at each observation point including time, global positioning system information (latitude and longitude), speed, course, altitude, road surface conditions (dry, chemically wet, critically wet, damp, ice, snow, and snow/ice), road surface temperature, road dew point temperature, relative road surface humidity, road surface friction, road surface ice percentage, road surface water film height, ambient temperature, and ambient humidity (refer to Appendix A). By the definition of the road surface condition in MARWIS [9], chemically wet refers to conditions wherein snow removal chemicals are applied to prevent ice formation, severe wet refers to conditions at the point of ice formation, and damp refers to a thin water film on the surface that does not significantly influence ice formation.

**Figure 2:**
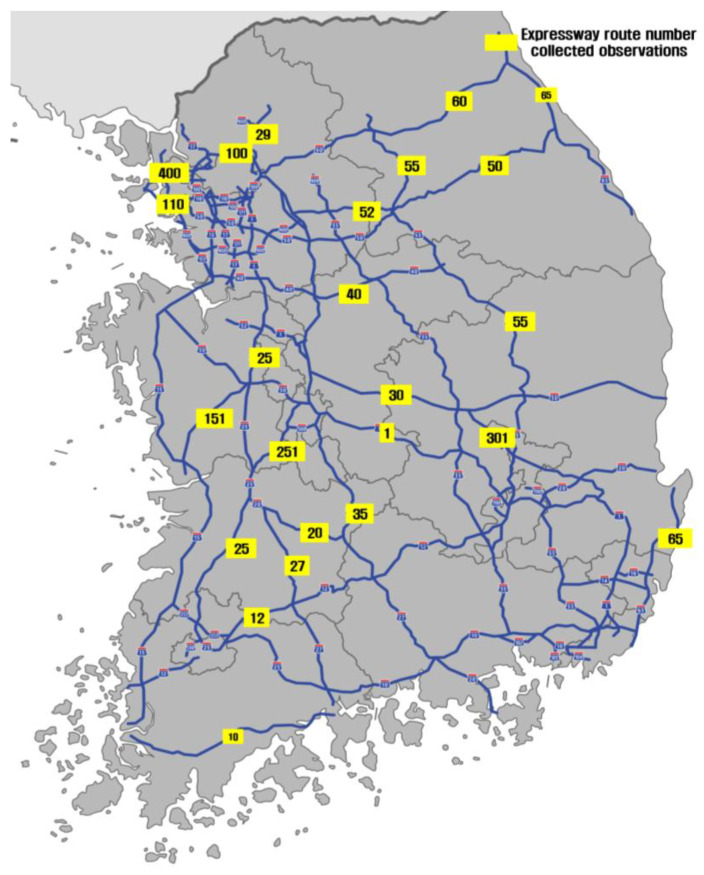
Data collection from various expressway routes [10].

Table 1 and Figure 2 show the expressways from which data were collected using a vehicle equipped with a sensor, as well as the sections and lengths of each route. A total of 4277.32 km were covered on 26 expressway routes. The total number of data points collected was 192,219. Among these, 186,917 (97.2%) were dry, 322 (0.2%) were damp, 189 (0.1%) were severe wet, and 4791 (2.5%) were snow/ice conditions.

### 4.2. Selecting Features and Responses

An item was selected as a feature of the machine learning model according to whether it was collected from low-cost detectors or open sources, as shown in Table 2. For the corresponding road surface state as its target label in this study, chemically wet and severe wet conditions, which have similar definitions according to MARWIS, were combined into one group (severe wet), and ice and snow were combined into the snow/ice group. Based on this, the groups (levels) applied for machine learning training were reclassified into four categories: dry, damp, severe wet, and snow/ice.

### 4.3. Data Visualization

**Figure 3:**
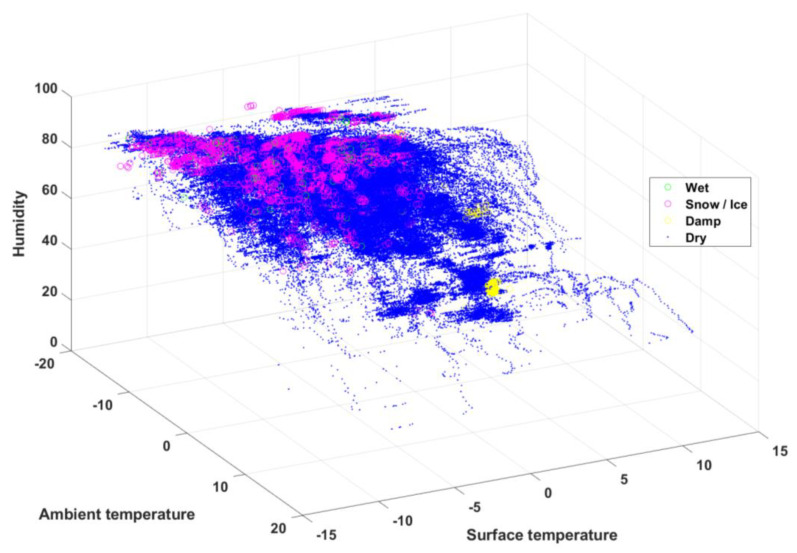
Scatter plot of humidity, ambient temperature, and road surface temperature.

**Figure 4:**
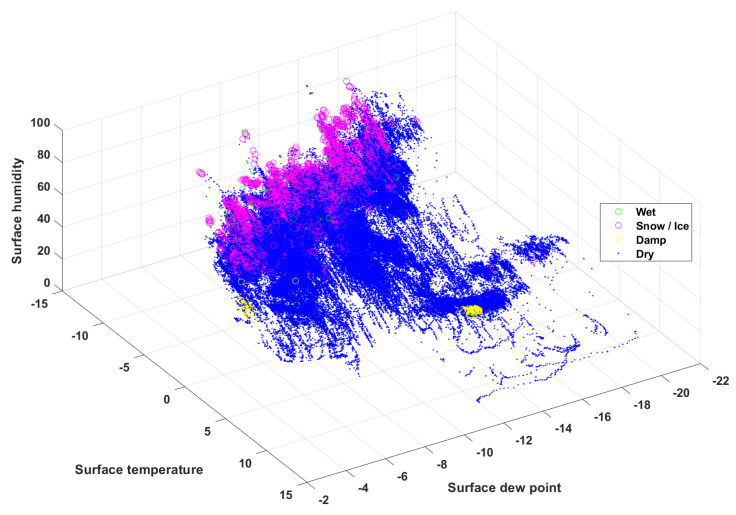
Scatter plot of surface humidity, surface dew, and surface temperature.

Figure 3 and Figure 4 show the data visualizations as a three-dimensional relationship with the quantitative raw data to help to visually and quickly identify general patterns between three of the eight features.

Figure 3 shows the data visualization between road surface temperature, ambient temperature, and ambient humidity with each road surface condition. The data for severe wet and snow/ice conditions are predominantly distributed at high humidity and low ambient and road surface temperatures. In particular, the snow/ice condition is distributed at higher humidity levels than the severe wet condition under the same ambient and road surface temperatures. Figure 4 shows the data visualization between road surface temperature, relative humidity of the road surface, and dew point on the road surface for each road surface condition. The snow/ice and severe wet conditions are distributed mostly in road surface regions with higher humidity and have a slightly higher dew point than that associated with dry conditions. Therefore, the weather features related to the road surface and the atmosphere show distinct trends for each road surface condition.

## 5. Support Vector Machine (SVM) Concept and Learning Methods

### 5.1. SVM Concept

Among supervised machined learning algorithms, SVM is a binary linear classification model that determines the group to which specific data belong; this is accomplished by finding a hyperplane that can divide a D-dimensional space into D-1 dimensions. SVM can also be efficiently applied to nonlinear classification, owing to its high-dimensional feature spaces. Furthermore, the kernel function allows for its extension to multi-class classification as well. It has been successfully applied to solve real-world problems in various fields [11,12].

**Figure 5:**
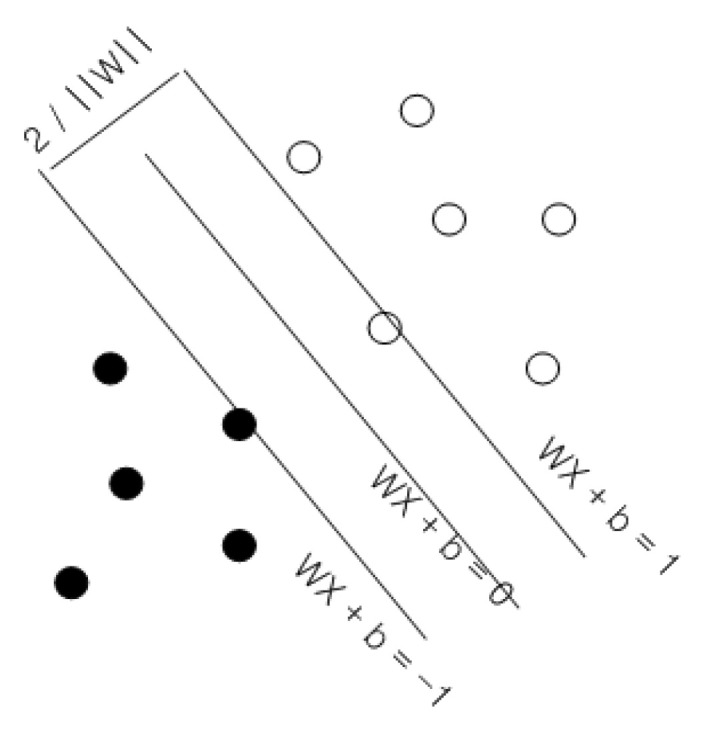
Decision boundary that maximizes the margin (group 1: black circles, group 2: white circles).

SVM is an algorithm that maximizes the margin (the distance between hyperplanes passing through each support vector) of the hyperplane that classifies two groups (1 and −1) (Figure 5). Furthermore, the hyperplane separates the data into two groups and satisfies the following condition:

(1) WX + b = 0

where W is the normal vector of the hyperplane and X is the support vector of the hyperplane, which is the closest data to the hyperplane from each group.

The SVM used in this study can handle both linear and nonlinear cases simultaneously, and it adopts a soft-margin classification technique that allows for a degree of misclassification for generalization. This method also applies kernel functions to convert features that are not linearly separable into high-dimensional feature spaces to enable linear separation. The following formula represents the objective function and constraints to maximize the weights (W) and bias (b) of the hyperplane using both a kernel function and a soft margin [11,12,13,14].

(2) $$\mathrm{argmax}\;\left(\alpha \right)\overset{B}{\underset{n-1}{\sum }}\alpha_{n}-\frac{1}{2}\overset{N}{\underset{n,m=1}{\sum }}\alpha_{n}\alpha_{m}t_{n}t_{m}k\left(X_{n},\;X_{m}\right)\;\mathrm{subject}\;\mathrm{to}\;\sum_{n=1}^{N}\alpha_{n}t_{n}=0\;\mathrm{and}\;0\;\leq \;\alpha_{n}\;\leq \;C\;\mathrm{for}\;\mathrm{all}\;n$$

(3) $$W=\overset{N}{\underset{n=1}{\sum }}\alpha_{n}t_{n}X_{n},\;b\;=\;{t_{n}}^{\prime }\;-\;\overset{N}{\underset{m=1}{\sum }}\alpha_{m}t_{m}k\left(X_{m},\;{X_{n}}^{\prime }\right)$$

where $\alpha_{n}$ is the Lagrange multiplier of the nth data—the label of the nth data (1 or −1), C is a cost parameter that controls overfitting, $k\left(X_{n},\;X_{m}\right)\;=\;\varphi {\left(X_{n}\right)}^{T}\varphi \left(X_{m}\right)$ is a kernel function, and $\varphi \left(•\right)$ is a transition function. Additionally, the kernel functions in this study are the most applied in the field and are listed in Table 3.

While SVM is successful in assigning specific groups, as a non-probabilistic classifier, it has the drawback of not providing reliable information on the probability of new data belonging to certain groups. Also, considering the uncertainty, it could be much more beneficial to provide the probability of belonging to a specific group based on the given data rather than simply providing the assigned result. Thus, the classification scores obtained using the trained SVM were used to calculate the posterior probability belonging to each specific group by applying the sigmoid function. SVM can be used to analyze whether severe wet, snowy, and icy road surface conditions, which are directly related to accidents on slippery roads, are more likely to occur in certain environments. Such information can enable drivers or road users to know in advance about the possibility of dangerous road surface conditions in certain environments, allowing them to take precautionary measures against road accidents by providing danger warnings.

### 5.2. SVM Training Method

Out of 768,873 data points, 70% were randomly allocated to training data and the remaining 30% to test data. The four group labels were dry, damp, wet, and snow/ice. Standardization was performed to eliminate the negative impact caused by the range differences between independent variables. The objective function (Equation (3)) was optimized using the iterative single-data algorithm or sequential minimal optimization.

To train the multi-class classification model using the SVM binary learner, two common learner designs were considered. One was the one-vs-one learner design, where one group was positive, another group was negative, and the remaining groups were ignored; the other was the one-vs-all learner design, where one group was positive and all remaining groups were negative. The optimal learner was selected, i.e., the one with the lowest generalized classification error in a five-fold cross-validation. Additionally, the parameters of Gaussian and polynomial kernel functions, which are hyperparameters, and the parameter (C) that controls overfitting were also optimized to minimize loss through five-fold cross-validation.

To evaluate the classification accuracy on the training data and the generalization performance on the test data for each multi-class classifier, a confusion matrix was created that compared the predicted values to the actual values, and the percentages of predictions that differed from the actual values were determined.

## 6. Results and Implications

### 6.1. Model Training Results

**Figure 6:**
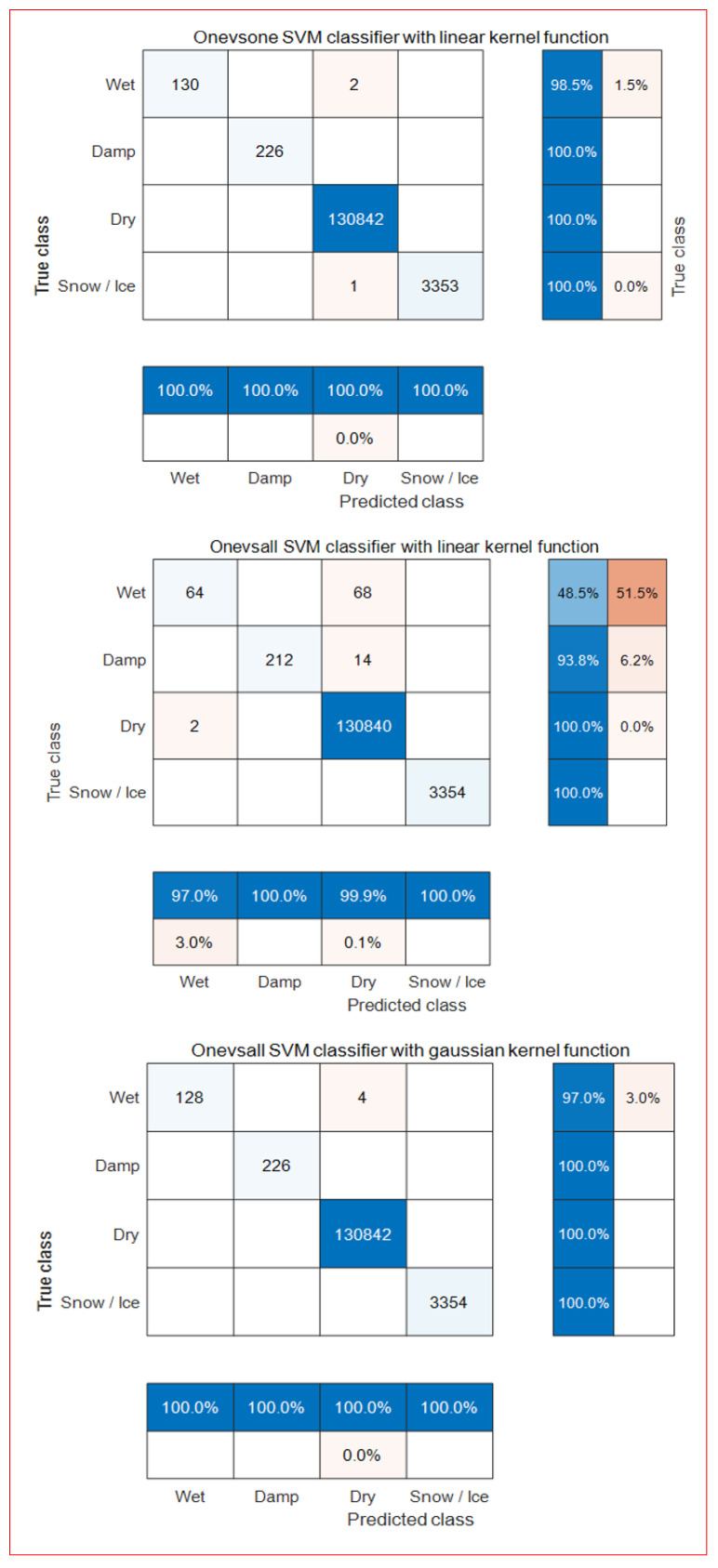

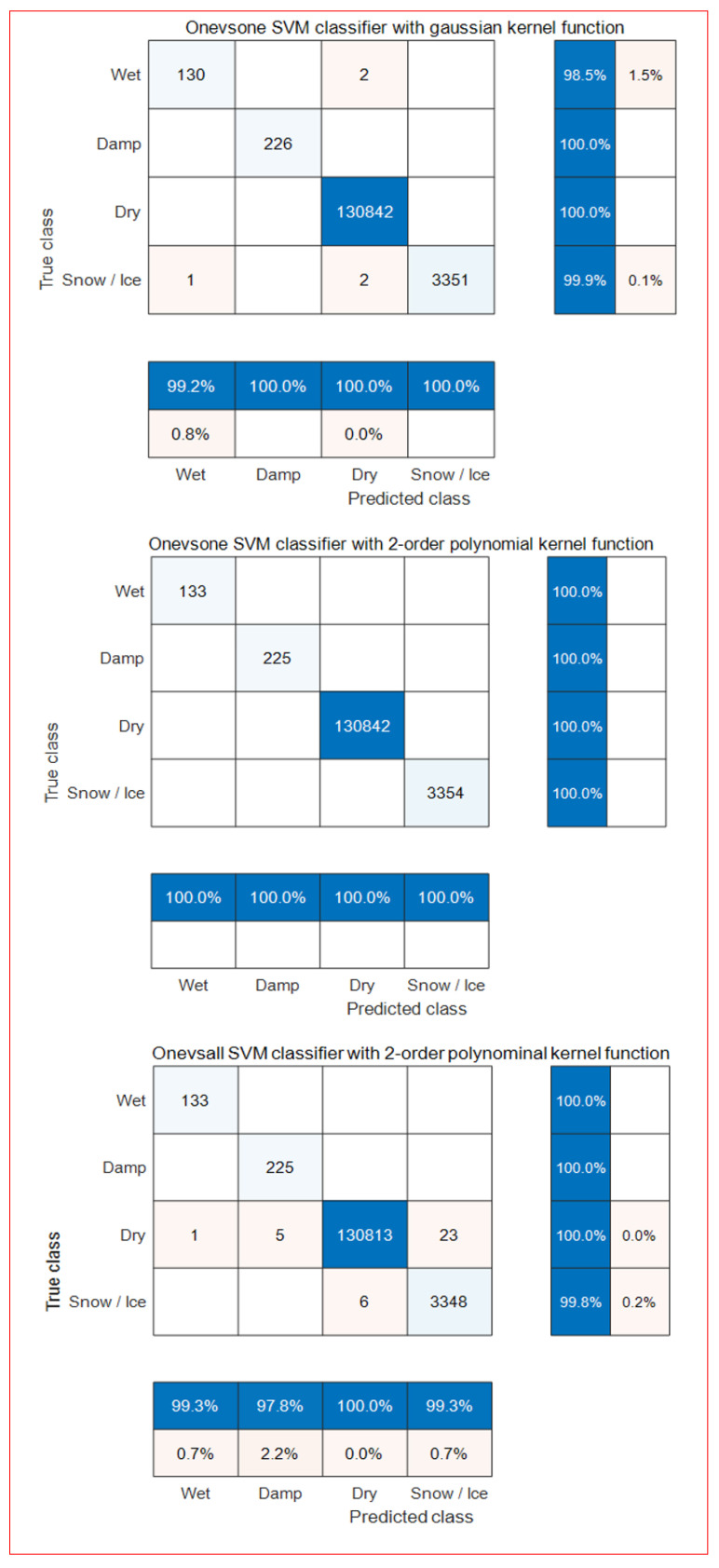
Confusion matrix by each trained SVM classifier.

Figure 6 shows the accuracy of each predicted class and true class using the hyperparameters of the optimized kernel function (γ), C, and the weights of the hyperplane based on the training data for each type of learner. Here, the accuracy of each predicted class is the ratio of correctly predicted road surface conditions to the total number of predictions made, and this information is presented at the bottom. The accuracy of each true class is the ratio of correctly predicted road surface conditions to those that were not accurately predicted, and this information is presented on the far right.

The classification errors of linear and nonlinear classifiers, excluding the one-vs-all linear learner, were below 3%, accurately classifying road surface conditions. The SVM classifier with a one-vs-one second-degree polynomial kernel function achieved the most accurate classification. Furthermore, the one-vs-one learning method was more accurate than the one-vs-all learning method in classification.

Although the accuracy of the SVM classifier using a second-order polynomial kernel function was 100%, the generalization performance of the road surface condition classification was evaluated using test data with a one-vs-one linear learner and a nonlinear learner of the Gaussian and the second-order polynomial kernel function.

### 6.2. Evaluation of Model Generalization Performance

The generalization performances of the three multi-class classifiers optimized with the training data were evaluated by applying test data through a confusion matrix.

**Figure 7:**
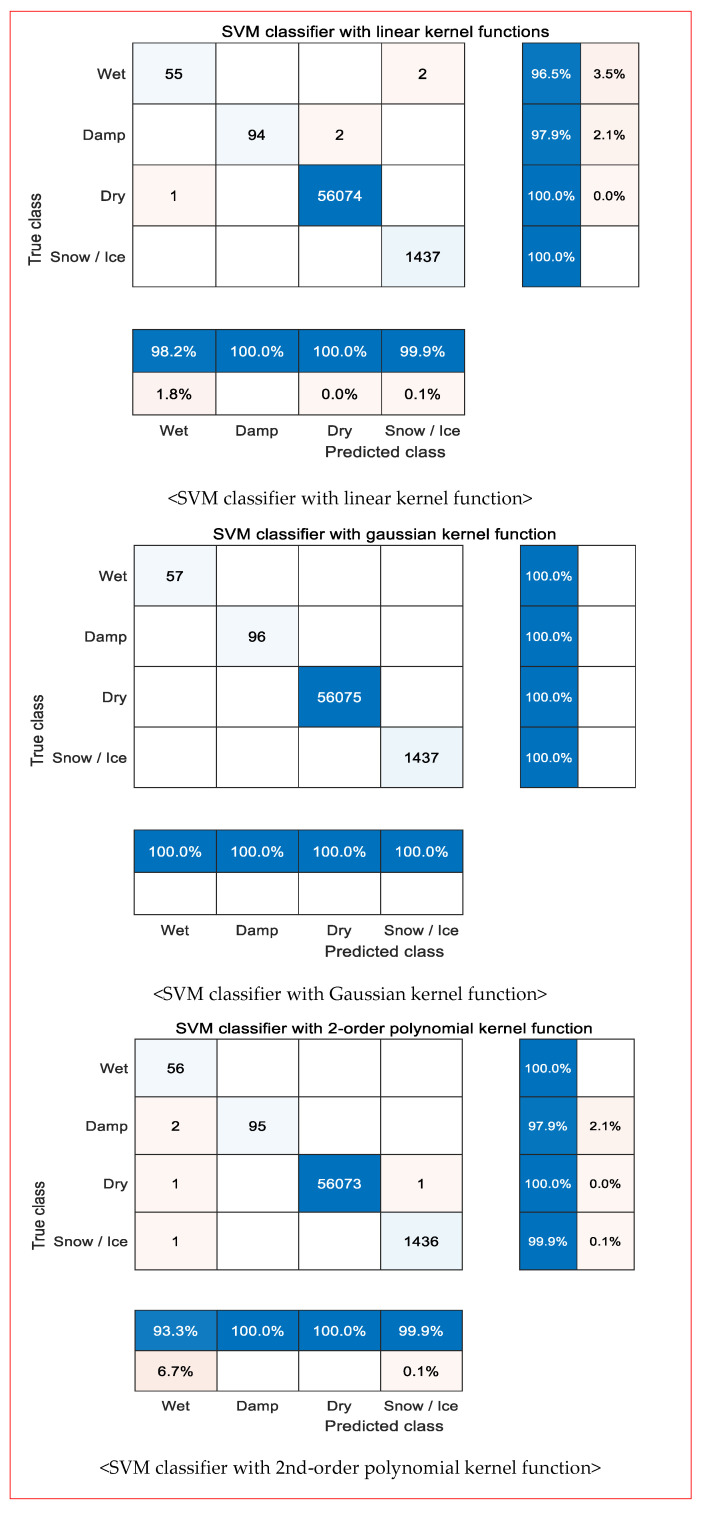
Estimating generalization performances of each SVM classifier using a confusion matrix.

As shown in Figure 6, Figure 7 displays the accuracy and error rates of each multi-class classifier for each prediction class (at the bottom) and true class (on the far-right side). The overall error rate of the three multi-class classifiers was less than 4%. Specifically, the accuracy of the multi-classifier with a Gaussian kernel function was 100%. By contrast, the generalization accuracy of the multi-class classifier with a second-degree polynomial kernel function was somewhat lower than the accuracy based on the training data.

**Figure 8:**
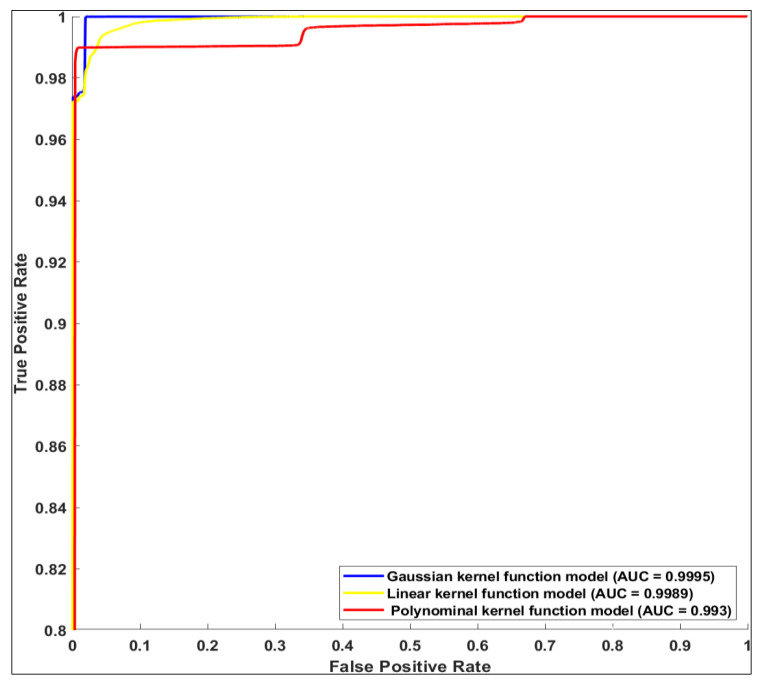
ROC curves for the SVMs.

In addition to the confusion matrices, by plotting the receiver operating characteristic (ROC) curve with the area under the ROC curve (AUC), we evaluated the performances of the models on the test data. Figure 8 shows that the multi-classifier with a Gaussian kernel function was the best performance, followed by the linear kernel function and the polynomial kernel function.

### 6.3. Posterior Probability Distributions

**Figure 9:**
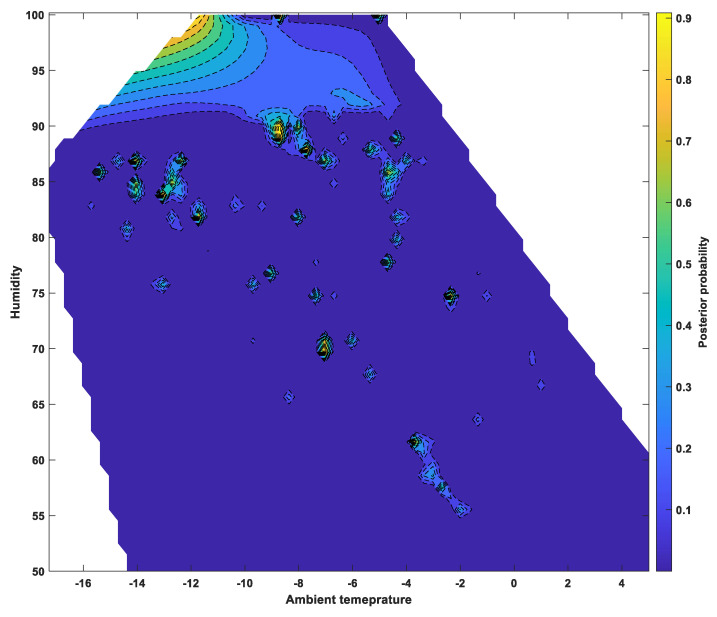
Posterior probability of a dangerous surface condition under ambient temperature and humidity.

**Figure 10:**
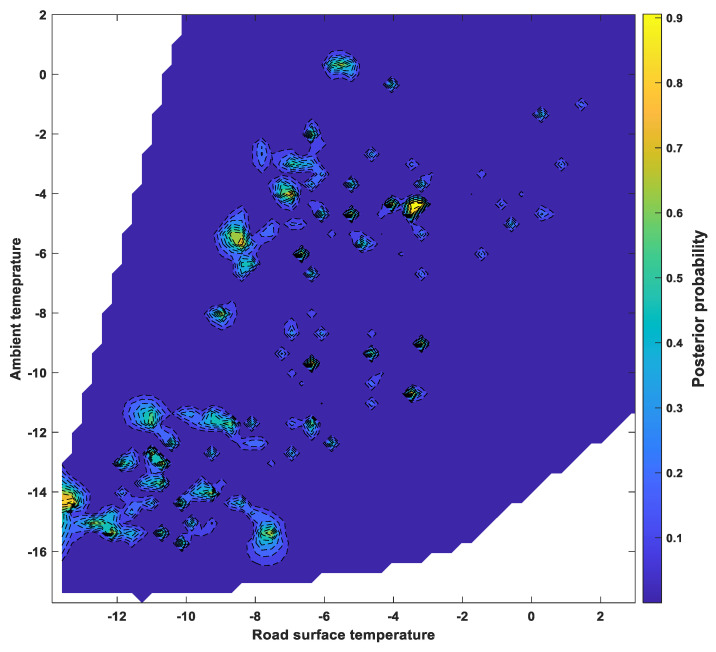
Posterior probability of a dangerous surface condition under ambient temperature and surface temperature.

**Figure 11:**
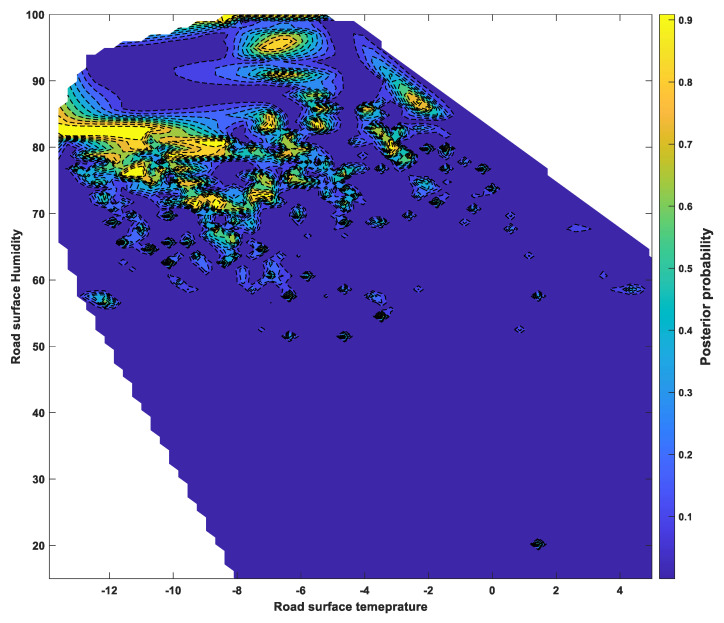
Posterior probability of a dangerous surface condition under surface temperature and surface humidity.

The classification scores determined by SVM using a one-vs-one Gaussian kernel function were applied to a sigmoid function to calculate the posterior probability of the occurrence of a “dangerous road surface condition,” which refers to severe wet, snowy, and or icy conditions and is directly related to traffic accidents. The posterior probabilities, calculated for each group, were summed to show the probability of a dangerous road surface condition under various environmental factors, as shown in Figure 9, Figure 10 and Figure 11. Figure 9 shows the ambient temperature and humidity, while Figure 10 shows the relationship between ambient temperature and road surface temperature. Figure 11 shows the probability of dangerous road surface conditions according to the road surface temperature and pavement moisture conditions.

The likelihood of hazardous road surface conditions was found to be higher at air temperatures below 0 °C and humidity above 50%, or at air temperatures below 0 °C and road surface temperatures below 5 °C. Furthermore, there was a high likelihood of hazardous road surface conditions when the road surface temperature was below 5 °C and the road surface moisture was above 50%.

The posterior probabilities showed that it is possible to analyze certain ambient and road surface weather conditions that correspond to a high probability of hazardous road surface conditions.

### 6.4. Implications

The data-driven learning model demonstrated accuracy in predicting road surface conditions; it was comparable to cost and advanced detectors. In addition, the classifier and these posterior probability distribution results might be highly useful for providing slippery road surface warnings compared to directly preventing slippery accidents. According to Lee et al. [15], Jeon et al. [16], and two analyses by the British Transportation Research Institute (TRL) [17,18], the positive effects of traffic safety information on speed reductions and driver behavior have been demonstrated, and significant decreases in road accidents have been found to be associated with these speed reductions. Thus, we expect that, if danger warnings are provided to drivers in advance, speed reductions or detours can be induced and slippery accidents can be indirectly prevented. Also, if warnings are provided to road management, they may take action to prevent slippery roads earlier.

## 7. Conclusions

This study evaluated the accuracy and generalizability of four road surface condition prediction models by utilizing SVM, a binary classifier using a kernel function that can efficiently apply linear and nonlinear classifications. In addition to evaluating the potential of the data-driven learning model, the posterior probability of belonging to each group was presented using the classification score calculated by the trained SVM.

The data used for building the learning model were collected between December 2022 and March 2023 using survey vehicles, including atmospheric and road surface weather-related data as well as road surface conditions. The data were preprocessed, including standardization of different characteristics among explanatory variables by normalization. Then, 70% of the preprocessed data were randomly allocated as training data, while the remaining 30% were used as test data. The training and validation processes were performed, and the accuracy and generalization were evaluated using a confusion matrix.

For the multi-class classifier, one linear and two nonlinear models (Gaussian and second-degree polynomial kernel functions) were considered, and the one-vs-one or one-vs-all learning method was considered. Training was conducted to determine the optimal hyperparameters and weight values for each model. The results showed that the error between the true class and the predicted class was below 3%, demonstrating a certain level of accuracy. Specifically, it was observed that the one-vs-one multi-class classifier using the second-degree polynomial kernel function had the highest accuracy among the classifiers, but its generalization performance was lower than its accuracy on the training data. The multi-class classifier of the Gaussian kernel function exhibited the highest generality performance. The posterior probabilities for dangerous road surface conditions, including severe wetness, snow, and icy conditions, were also evaluated. The results showed that it is possible to analyze certain atmospheric and road surface conditions that correspond to a high probability of hazardous road surface conditions. The classifier and posterior probability distributions can be sufficiently used in advance to predict slippery road conditions.

Therefore, we conclude that, if atmosphere and road surface-related weather data can be secured, it is possible to build a data-driven learning model to predict slippery road conditions comparable to cost detectors.

This study only considered the SVM to demonstrate the possibility of accurate road surface condition prediction under adverse winter weather conditions using a data-driven learning model. To develop a model with excellent predictive performance in the future, different machine learning models need to be applied. Furthermore, additional variables for road segments based on the characteristics of road pavement need to be considered, because the thermal properties of the road surface can significantly affect the road surface temperature.

## Figures and Tables

**Table 1:** Data collection sections [10].

Expressway Number	Section	Length of Section (km)
1	Guseo IC (Interchange)–Yangjae IC	416.05
10	Daejeo IC–Seosuncheon IC	273.1
	Haeryong IC–Seoyoungam IC	
12	Goseo JC (Junction)–Okpo JC	181.9
	Muan Airport IC–Unsu IC	41.3
15	Geumcheon IC–Jukrim JC	340.8
20	Iksan JC–Jangsuri JC	60.0
	Pohang IC–Palgongsan IC	69.4
25	Cheonan JC–S. Nonsan TG	82.0
25	Nosan JC–S. Suncheon	194.2
27	E. Suncheon IC–Wanju JC	117.8
29	S. Guri IC–Sinbuk IC	50.6
	Sohol JC–Yangju IC	
30	Dangin JC–Yuseong JC	278.6
	Cheongju JC–Youngduk IC	
35	Hanam JC–Namyi JC	332.5
	Biryong JC–Tongueong IC	
40	Jecheon JC–W.Pyeongtaek JC	126.9
45	Yangpyeong IC–Naeseo IC	301.7
50	Seochang JC–Gangneung JC	234.4
52	Gyeonggi-Gwangju JC–Wonju JC	59.65
55	Geumho JC–Chuncheon IC	278.5
55	E.Daegu JC–Daedong JC	82.0
60	Yangyang JC–Gangil IC	150.2
65	Geundeok IC–Sokcho IC	122.16
	Janggeom IC–S.Pohang IC	53.7
65	New Haeundae Station–Janggeom IC	47.2
100	Uijeongbu IC–Uijeongbu IC(Ilsan direction)	128.8
110	Neunghae IC–Sammak IC	26.7
151	E. Seocheon IC–S.Gongju JC	61.4
251	Hoedeok JC–Nosan JC	54.0
301	Nakdong JC–Yeongcheon JC	93.96
400	Bongdam IC–Dongtan JC	17.8

**Table 2:** Selected features and response.

Features	Classification (Response)
Road surface temperature (°C) Road dew point temperature (°C) Relative road surface humidity (%) Road surface friction Road surface water film height (mm) Ambient temperature (°C) Ambient humidity (%)	Dry Damp Severe wet Snow/ice

**Table 3:** Kernel functions [11,12].

Kernel Function	Equation
Linear	$k\left(X_{n},\;X_{m}\right)\;=\;{X_{n}}^{T}X_{m}$
Gaussian	$k\left(X_{n},\;X_{m}\right)\;=\;\exp\{-\gamma {\left(X_{n}-X_{m}\right)}^{T}\left(X_{n}-X_{m}\right)\}$
Polynomial	$k\left(X_{n},\;X_{m}\right)\;=\;{\left(1+{X_{n}}^{T}X_{m}\right)}^{\gamma }$

**Figure A1:**
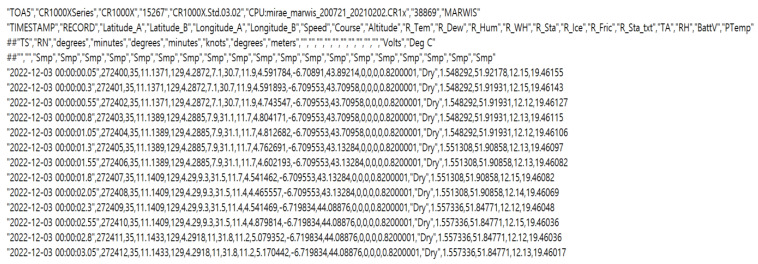
A piece of raw data collected by MARWIS.

## Data Availability

Data are contained within the article.

## Appendix A

A piece of raw data collected by MARWIS is shown in the figure below.
